# Luteolin Mitigates D-Galactose-Induced Brain Ageing in Rats: SIRT1-Mediated Neuroprotection

**DOI:** 10.1007/s11064-024-04203-y

**Published:** 2024-07-11

**Authors:** Reham L Younis, Rehab M El-Gohary, Asmaa A Ghalwash, Islam Ibrahim Hegab, Maram M Ghabrial, Azza M Aboshanady, Raghad A Mostafa, Alaa H. Abd El-Azeem, Eman E. Farghal, Asmaa A.E. Belal, Haidy Khattab

**Affiliations:** 1 Medical Physiology Department, Faculty of Medicine, Tanta University, Tanta, Egypt; 2 Medical Biochemistry Department, Faculty of Medicine, Tanta University, Tanta, Egypt; 3 Bio-Physiology Department, Ibn Sina National College for Medical Studies, Jeddah, Saudi Arabia; 4 Anatomy & Embryology Department, Faculty of Medicine, Tanta University, Tanta, Egypt; 5 Clinical and Chemical Pathology Department, Faculty of Medicine, Tanta University, Tanta, Egypt; 6 Medical Pharmacology Department, Faculty of Medicine, Tanta University, Tanta, Egypt; 7 Neuropsychiatry Department, Faculty of Medicine, Tanta University, Tanta, Egypt

**Keywords:** Brain ageing, Luteolin, Mitochondrial dysfunction, D-galactose, AGE/RAGE axis, SIRT1

## Abstract

Luteolin is an essential natural polyphenol found in a variety of plants. Numerous studies have supported its protective role in neurodegenerative diseases, yet the research for its therapeutic utility in D-galactose (D-gal)-induced brain ageing is still lacking. In this study, the potential neuroprotective impact of luteolin against D-gal-induced brain ageing was explored. Forty rats were randomly divided into four groups: control, luteolin, D-gal, and luteolin-administered D-gal groups. All groups were subjected to behavioural, cholinergic function, and hippocampal mitochondrial respiration assessments. Hippocampal oxidative, neuro-inflammatory, senescence and apoptotic indicators were detected. Gene expressions of SIRT1, BDNF, and RAGE were assessed. Hippocampal histopathological studies, along with GFAP and Ki67 immunoreactivity, were performed. Our results demonstrated that luteolin effectively alleviated D-gal-induced cognitive impairment and reversed cholinergic abnormalities. Furthermore, luteolin administration substantially mitigated hippocampus oxidative stress, mitochondrial dysfunction, neuro-inflammation, and senescence triggered by D-gal. Additionally, luteolin treatment considerably attenuated neuronal apoptosis and upregulated hippocampal SIRT1 mRNA expression. In conclusion, our findings revealed that luteolin administration attenuated D-gal-evoked brain senescence, improving mitochondrial function and enhancing hippocampal neuroregeneration in an ageing rat model through its antioxidant, senolytic, anti-inflammatory, and anti-apoptotic impacts, possibly due to upregulation of SIRT1. Luteolin could be a promising therapeutic modality for brain aging-associated abnormalities.

## Introduction

Aging is a multifactorial phenomenon that results in increasing molecular and physiological malfunctions. [1] Recently, oxidative stress (OS), mitochondrial dysfunction, inflammation, and apoptosis have been widely recognized as the primary factors contributing to the pathophysiology of the ageing process. [2] The most obvious manifestation of ageing in the human body is the ageing of the brain, causing cognitive impairment and hippocampal neurogenesis reduction. [3] Animal models have been used to study the pathophysiological mechanisms of brain ageing, but the most widely used model is the D-galactose (D-gal)-evoked brain ageing. [4] Giving animals a large dosage of D-gal for a long period (6–10 weeks) can cause brain ageing in a manner that is similar to human brain ageing in many regards. [5]

D-gal in high doses may impair the body’s ability to convert galactose into glucose, increasing aldose reductase activity and galactitol formation, causing oxidative injury to cells, and forming advanced glycation end products (AGEs) [4]. AGEs are heterogenous compounds derived when the reactive carbonyl group of reducing sugars reacts non-enzymatically with the amino groups of biomacromolecules like proteins, lipids, or nucleic acids through nucleophilic addition. [6] Recently, AGEs have been implicated in the pathophysiology of many aging-associated pathological circumstances. [4, 6] AGEs interact with their specific receptors (RAGEs) in various cell types and activate inflammatory signalling pathways, resulting in the overproduction of ROS and reduction in the antioxidant capacity. [7] ROS triggers oxidative damage to cellular proteins, lipids, and DNA, causing cellular dysfunction and apoptosis. Mitochondria are the primary generators of free radicals. Moreover, mitochondria are primarily impacted by ROS. The subsequent release of ROS, in a self-perpetuating loop, evokes mitochondria dysfunction and triggers cell death, consequently accelerating age-related changes. [8] This enhances the expression of bio-specific senescence-related β-galactosidase and genes (p16, p53, p19Arf, p21Cip1/Waf1). [9]

Intriguingly, methylglyoxal (M.G.) is a reactive carbonyl species produced endogenously in cells, primarily during glycolysis. Under normal conditions, the glyoxalase (GLO) system converts M.G. into non-toxic D-lactic acid, with GLO1 being the rate-limiting enzyme. [10] Biochemically, abnormal glycolysis or long-term consumption of foods that contain high amounts of M.G. causes the body’s clearance system to become overloaded, accumulating M.G. [11] By reacting with long-lived proteins, M.G. evokes severe cytotoxicity and tissue damage, resulting in irreversible crosslinking to AGEs, which bind to their RAGEs, augmenting ROS generation and activating nuclear factor kappa B. This then provokes exacerbation of pro-inflammatory cytokines, such as IL-1β and TNF-α, triggering chronic OS and neuro-inflammation, ultimately ending in brain senescence. [10] Therefore, activating the glyoxalase system is likely to protect the brain from oxidative damage elicited by D-gal.

Sirtuin 1 (SIRT1), a nicotinamide adenosine dinucleotide (NAD^+^)-dependent protein deacetylase, is implicated in regulating a variety of cellular events, including cell cycle, senescence, apoptosis, and metabolism by interacting with molecules involved in these processes, such as P53 [12]. Accumulating data indicates SIRT1 is a longevity factor that hinders the development of age-related brain dysfunction and neurodegenerative disorders. [13] Recent research has found that downregulating SIRT1 causes a premature senescence-like phenotype. Meanwhile, SIRT1 overexpression renders cells more resistant to senescence-related physiological changes [2], making it a viable target for delaying ageing.

A growing body of evidence suggests that the brain has the highest oxygen requirement and mitochondrial content in the body as well as a limited antioxidant system. As a result, it is particularly vulnerable to oxidative damage. [14] Accordingly, natural antioxidants possessing multiple health benefits have recently attracted attention as substitute medication, including luteolin. Luteolin is a naturally occurring flavonoid present in celery, green pepper, and chamomile. Luteolin has antioxidant, anti-inflammatory, anti-carcinogenic, and anti-apoptotic activities [15]. It also reduces microglia and modulates hippocampal-dependent spatial working memory in aged mice, improves hippocampal and cortical morphological changes, and plays a protective role in neurodegenerative diseases. [9] It was recently shown that luteolin dramatically reduced endoplasmic reticulum stress and neuro-inflammation in Alzheimer’s disease mice. [16] Despite this, further research is needed to determine whether luteolin impacts brain senescence-related mechanisms.

Several studies have suggested that luteolin has potential CNS advantages. [17] However, research into its potential impact on D-gal-induced brain senescence remains limited. Thus, this work sought to shed light on the effects of luteolin on experimental D-gal-induced brain ageing by tracking cell senescence, mitochondrial dysfunction, and apoptotic indicators, as well as histological and immunohistochemical findings.

## Materials and Methods

### Drugs and Chemicals

D-gal (CAS-No.: 59-23-4), luteolin (CAS-No.: 491-70-3), and most chemicals used, unless otherwise mentioned, were ordered from Sigma-Aldrich Chemicals Co. (St. Louis, MO, US). All reagents were of the reagent grade, and all were commercially available. All solutions were prepared daily.

### Animals

Forty male Wistar rats (8-week-old, 160–190 g) were purchased from the Animal Breeding Laboratory of the Faculty of Science, Tanta University (Tanta, Egypt). Rats were group-housed in well-ventilated wire mesh cages, 5 per cage, and maintained at ambient temperature and humidity under a 12-hour controlled light/dark cycle (light phase daily started at 8.00 am) within the Animal House of the Medical Biochemistry Department, Faculty of Medicine, Tanta university. Tap water and chow (EL-Nasr Chemical Company, Cairo, Egypt) were made available ad libitum throughout the experiment. Animals were acclimated to the housing environment for one week before the experiment. The experimental procedures were consistent with the guiding principles of the Institutional Animal Research Ethical Committee, Faculty of Medicine, Tanta University, Egypt (Permit code: 36264PR344/9/23).

### Experimental Plan Schedule

**Fig. 1:**
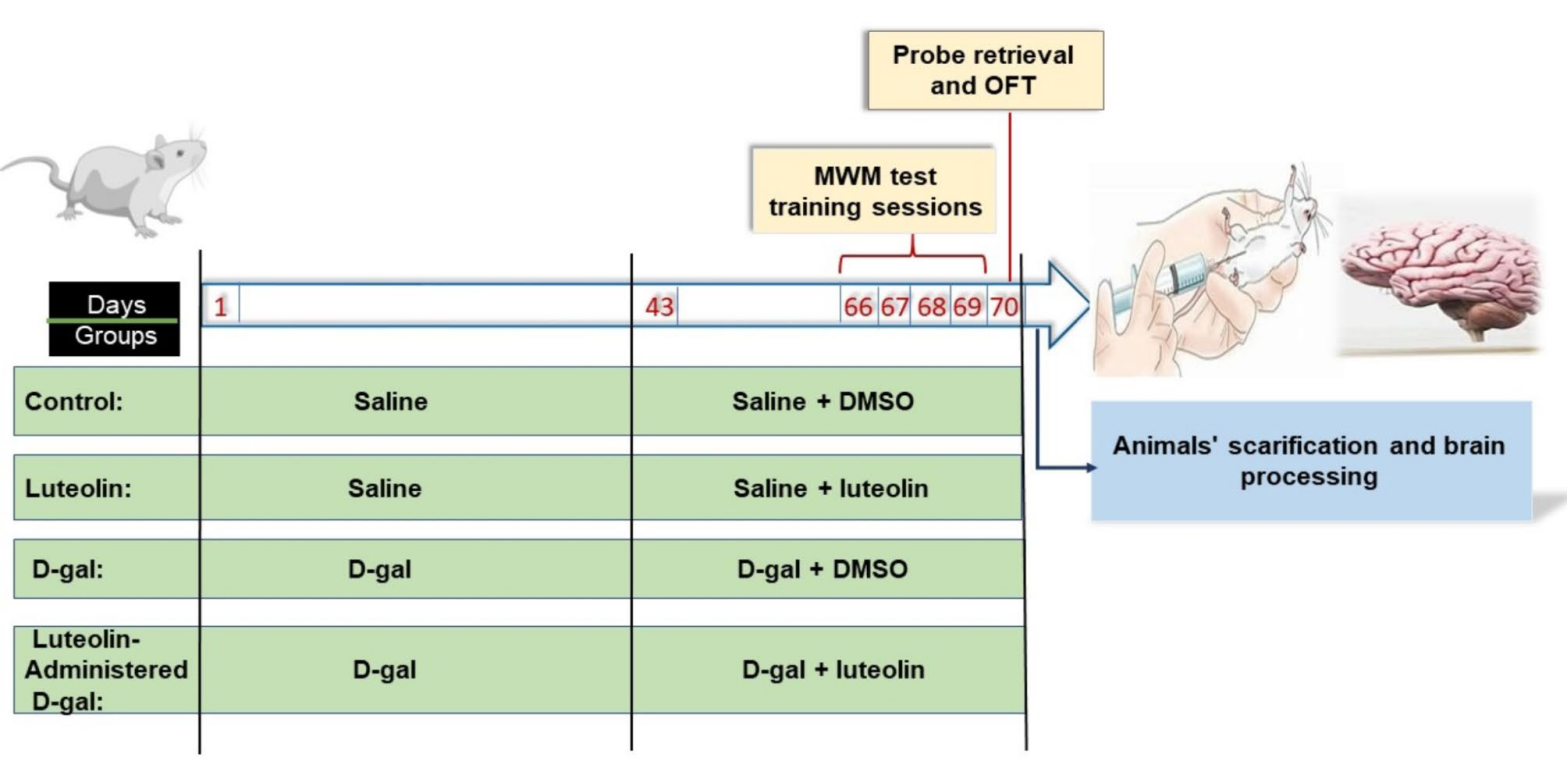
Schematic representation of the study protocol

Following the one-week acclimation period, rats were screened utilizing the Morris water maze (MWM) test to select the qualified rats that could reach the submerged platform within 90-s. The unqualified animals were banned from the study. [18] Forty qualified rats were randomly assigned into four groups of ten rats each, as ascribed in Fig. 1: **control (Ctrl) group**: rats received a daily subcutaneous (SC) injection of 0.2 ml of isotonic saline for ten consecutive weeks, coupled with a single daily intraperitoneal (IP) injection of dimethyl sulfoxide (DMSO) starting from the seventh week and for four successive weeks. **Luteolin group**: rats were treated as the Ctrl group except for a single daily IP injection of luteolin (80 mg/kg/day, dissolved in 1% DMSO) starting from the seventh week and for four successive weeks. **D-gal group**: rats were daily administered D-gal (150 mg/kg/day) via SC injection once a day for ten consecutive weeks, coupled with a single daily IP injection of DMSO starting from the seventh week and for four successive weeks. **Luteolin-administered D-gal group**: rats were daily administered D-gal (150 mg/kg/day) via SC injection once a day for ten consecutive weeks, coupled with a single daily IP injection of luteolin (80 mg/kg/day, dissolved in 1% DMSO) starting from the seventh week and for four successive weeks. The doses used in this work were based on published works. [19, 20] Throughout the work, rats were closely monitored daily. There were no animal deaths in any of the studied groups. Body weights were detected every Saturday, and body weight changes were calculated.

### Behavioural Assessments

Behavioural tests were carried out in the light phase of the day (between 8.00 and 15.00). All rats were subjected to the MWM test five days prior to the end of our experiment. Memory training sessions were held for four consecutive days, followed by the spatial probe test. Moreover, the open field test (OFT) was conducted after the probe trial, as depicted in Fig. 1.

#### Assessment of the Spatial Memory and Learning by MWM Test

The MWM test was conducted following a previously described protocol. [21] The MWM apparatus consisted of a circular water pool (120 cm in diameter and 50 cm in height). The apparatus was virtually divided into four quadrants and filled with water (22 ± 1 °C) at 30 cm depth. A circular platform (10 cm in diameter) was submerged 2 cm below the water level at the second quadrant centre. The procedure comprises navigation training trials and probe testing. During the memory training phase, each rat received two daily training sessions for four successive days. During the trials, rats were placed in the pool at any of the three start quadrants (excluding the second quadrant) facing the wall. Each rat was allowed to swim for 90 s to reach the submerged platform and settle on the platform for 15 s. During acquisition, If the rat failed to find the submerged platform within 90 s, it was gently directed to the platform and allowed to stay on it for 15 s to memorize its location. The probe test (retrieval test) was carried out the day after the last training trial. The platform was removed, and the rats could swim freely within the pool and search for it for 90 s. The results of this test relied upon the analysis of two distinct parameters; the first was the escape latency (EL), which was measured as the time each rat took to find out the platform. The second parameter was the frequency of passing through the previous platform position within the 90-s, defined as the number of platform crossings, and represented as an index of retrieval.

#### Assessment of the Locomotor and Exploratory Activity by the OFT

The OFT was conducted in coherence with a formerly described method. [22] The open field was constructed as a rectangular-shaped arena (81 × 28 cm); its floor was divided by black lines into equal squares. Rats were positioned for approximately 1 min in the open field central zone to be adapted. The time spent exploring the centre of the arena, as well as the total number of squares crossed by the animals in the 5 min time limit, are the variables for analysis in the OFT test.

### Tissue Sampling

Twenty-four hours after OFT, rats were weighed, anaesthetized with IP sodium pentobarbital (60 mg/kg), and, in turn, sacrificed by cervical decapitation. The rats’ skull vaults were dissected out; brains were carefully harvested, dipped in ice-cold saline, left to dry on filter papers, and weighed for calculation of brain index [Brain index = brain weight (mg)/body weight (g)]. In turn, brains were sagitally split into two hemispheres. Hippocampi were meticulously extracted from all the right hemispheres and split into two fractions. The first fraction was homogenized in an ice bath in phosphate-buffered saline (PBS,1/5 (w/v), 0.1 M, pH 7.4) and, in turn, centrifuged at 10,000×g for 10 min at 4 °C. The supernatant aliquot was maintained at − 80 °C till further biochemical assays. The second portion was used for isolation of the hippocampal crude mitochondrial fraction. It was homogenized on ice in a mitochondrial isolation buffer (0.01 M Tris–HCl, 0.0001 M EDTA-2Na, 0.01 M sucrose, 0.8% NaCl, pH 7.4). Being properly homogenized, the obtained homogenate was centrifuged for 10 min at 2,000 xg followed by an additional centrifugation of the gained supernatants for 20 min at 5,000 xg. Total protein content was detected in the hippocampal tissue homogenate and the crude mitochondrial fraction, according to Lowry et al. [23] On the other hand, the hippocampi harvested from all the left hemispheres were fixed in 10% (v/v) formal saline for the histopathological and immunohistochemical studies.

### Biochemical Assays

#### Assessment of Senescence-Related Biomarkers

First, the previously prepared hippocampal homogenate was investigated for GLO1 enzymatic activity following a published procedure with slight modifications. [24, 25] The reaction was initiated by adding the tissue lysate to the assay mixture (2 mM of MG, 1 mM of reduced glutathione (GSH), and 100 mM of KH_2_PO_4_ in pH 6.6). Increased absorbance at 240 nm wavelength indicated the formation of S-D-lactoylglutathione. Each unit of GLO1 activity could be defined as the construction of 1 µmol of S-D-lactoylglutathione/min/mg tissue protein. Moreover, the hippocampal AGEs and P21 levels were immunoassayed using rat-specific ELISA kits (My BioSource Inc.; cat#MBS261131and Biorbyt Cambridge UK; cat#orb780018, respectively) according to the manufacturer’s guidelines.

#### Assessment of the Cholinergic Function

The hippocampal acetylcholine (Ach) level was assessed using a rat-specific microplate assay kit (My BioSource Inc.; cat#: MBS282680) according to the manufacturer’s protocols. In addition, the hippocampal acetylcholine esterase (AChE) enzymatic activity was determined at a 410 nm wavelength using a colourimetric assay kit (Abcam; cat# ab138871). In brief, the technique relied on the measurement of ACh conversion into thiocholine, which subsequently interacts with 5,5-dithiobis-2-nitrobenzoic acid (DTNB), constructing a coloured product, which is commensurate to the specimens’ AChE activity. [26]

#### Assessment of the Hippocampal Redox Homeostasis and Antioxidant Capacity

Using a colourimetric assay kit (Bio-diagnostic, Giza, Egypt, cat#: MD2529), the hippocampal redox status was assessed by measuring thiobarbituric acid reactive substances (TBARS) at 534 nm wavelength. The following technique comprised the reaction of thiobarbituric acid (TBA) and malondialdehyde (MDA) existing in the hippocampal homogenate in an acidic medium at 95 °C for 30 min, producing a pink-coloured product, which is commensurate to the specimens’ MDA content. [27] In addition, xanthine oxidase (XO) activity was assessed spectrophotometrically at 290 nm wavelength. The technique was based on the XO-catalyzed oxidation of xanthine into uric acid, which is directly proportional to XO catalytic activity. [28, 29] On the other hand, the hippocampal superoxide dismutase (SOD) activity was assessed colourimetrically based on a previously depicted technique. [30]

#### Assessment of Neuro-Inflammatory Biomarkers

The hippocampal tumour necrosis factor-α (TNF-α) and interleukin-1β (IL-1β) were immunoassayed using Rat specific ELISA kits (Cusabio, China, cat# CSB-E11987r and CSB-E08055r) based on the manufacturer’s protocol.

#### Assessment of the Efficacy of the Hippocampal Mitochondrial Respiration

Citrate synthase (CS) catalytic activity was assessed in the prepared mitochondrial extract following a previously stated protocol. [31] In brief, the reaction mixture (0.1 mM DTNB, 0.5 mM oxaloacetate, 50 mM EDTA, 0.31 mM acetyl coenzyme A, 5 mM triethanolamine hydrochloride, and 0.1 M Tris–HCl) was prepared and preheated for 5 min at 30 °C. The mitochondrial extract was added, and the enzymatic activity was recorded colourimetrically at 412 nm wavelength. In addition, the electron transport chain (ETC) complex I, NADH-Ubiquinone oxidoreductase, enzymatic activity was spectrophotometrically assessed using Birchmachin et al. [32] methodology. This protocol relied upon the decrease in the absorbance at 340 nm wavelength because of the enzyme-catalyzed NADH oxidation.

### Molecular Assay

According to the manufacturers’ guidelines, the total RNA was extracted from the frozen hippocampal tissue using the Gene JET RNA purification kit (Thermo Fisher Scientific, Waltham, USA; Cat #K0731). Concentration and purity of the gained RNA were assessed using the Analytik Jena NanoDrop spectrophotometer and ultimately kept at − 80 °C till reverse transcribed. An aliquot of 5 µg of RNA was reverse transcribed into complementary DNA (cDNA) with the aid of the Revert Aid H Minus Reverse Transcriptase kit (Thermo Fisher Scientific, Waltham, USA; Cat # EP0451). cDNA was used as a template for the real-time polymerase chain reactions (qRT-PCR) using INTRON SYBR-Green master mix (Biotechnology, Korea) in the StepOne Plus system (Applied Biosystem, USA). Quantification of the target mRNA transcripts was accomplished relative to the constitutive gene β-actin as an internal control. The relative gene expression was analyzed utilizing the 2^−ΔΔCt^ method. [33] The nucleotide sequences of the different gene-specific primers are listed in Table 1. They were all designed by the Primer3Plus tool (https://www.primer3plus.com/).

**Table 1:** Primer list for the qRT-PCR

Gene	Forward primer (5’-3’)	Reverse primer (5’-3’)
RAGE	CTACCTATTCCTGCAGCTTC	CTGATGTTGACAGGAGGGCTTTCC
BDNF	TGTCCGAGGTGGTAGTACTTCATC	CATGCAACCGAAGTATGAAATACC
Caspase 3	GCAGCAGCCTCAAATTGTTGACTA	TGCTCCGGCTCAAACCATC
SIRT1	TGTTTCCTGTGGGATACCTGA	TGAAGAATGGTCTTGGGTCTTT
β- Actin	GGCTGTGTTGTCCCTGTAT	CCGCTCATTGCCGATAGTG

### Histopathological Studies

Hippocampal specimens were first fixed in 10% (v/v) formal saline, dehydrated in ascending alcohol grades, then treated with xylol, and eventually immersed in paraffin. 5 μm-thickened sections were cut by a rotatory microtome (Leica, USA) to be subjected to:

#### Haematoxylin and Eosin Staining (H&E) for Evaluation of the Cytomorphological Characteristics

#### Immunohistochemical Staining for Evaluation of the Glial Fibrillary Acidic Protein (GFAP) and Ki67 Immunoreactivity

• For GFAP; sections were washed in PBS several times and treated for 30 min with 3% (dilution from 30%) H_2_O_2_ then washed with PBS before applying the primary antibody. The primary antibody (GFAP-rabbit monoclonal antibody) was diluted 1:20 in PBS and added and incubated for 1 h. The primary antibody was omitted before adding the secondary antibody biotin anti-rabbit, which was diluted 1:20 in PBS containing 1% bovine serum albumin (Abcam, Cambridge, USA; cat# ab207165) for 30 min.
• For Ki 67; Tissue sections were incubated in 20% methanol containing 0.3% H_2_O_2_ with 0.1% sodium azide to suppress the activity of endogenous peroxidase. Then, tissue sections were placed in an antigen retrieval solution (0.01 M citrate buffer, pH 6.0) for 15 min in a microwave oven at 100 C at 600 W. Then, Ki 67 rabbit monoclonal antibody (Abcam, Cambridge, USA; cat# ab16667) was applied to the sections at dilutions of 1:50. Incubations with primary antibodies lasted for 1 h at RT. After that Sections were incubated with biotinylated goat anti-rabbit IgG (dilution 1:200) (the secondary antibody) and AB complex.
• Finally, the slides of both stains were washed in distilled water, dried and placed in xylene for 5 min and mounted with a mixture of distyrene, a plasticizer and xylene (DPX) to preserve stain. GFAP stain revealed brownish colouration of the processes of astrocytes (dendrites and spines) whereas, Ki 67 stain showed brown coloured nuclei in the S- phase of cell cycle.

### Morphometric Analysis

Photomicrographs were taken using an Olympus BX43 light microscope (Tokyo, Japan). Image analysis was conducted using the software (Image J) program (1.46a, NIH, Bethesda, MD, USA). Ten randomly selected non-overlapping fields (x200) from each slide of each rat were examined.

### Statistical Methods

For statistical analyses, the Statistical Package for Social Science (SPSS) (IBM, Armonk, NY, USA; version 23) was used. Multiple comparisons were performed by the One-way ANOVA with Tukey’s ad hoc test. Results were presented as means ± SD. The correlation between the studied parameters was calculated using Pearson’s correlation coefficient. A level of *P* < 0.05 was considered significant.

## Results

### Effect of Luteolin Treatment on Behavioural Tests in D-Gal-Triggered Brain Ageing

Table 2 revealed that the Morris water maze and open field tests were used to evaluate spatial learning and memory with the possible mitigating role of luteolin treatment. Our results revealed that the D-gal-treated rats had memory and cognitive aberration, as indicated by the longer escape latencies and reduced number of platform crossings compared with those of the control rats. In contrast, the D-gal/luteolin group had significantly shorter latencies and more crossed platforms in contrast to those in the D-gal one, which was also insignificant versus the Ctrl group.

Simultaneously, in the open field test, the D-gal group displayed a remarkably reduced total number of rearing and squares crossed in 5 min compared to the Ctrl group. Conversely, Luteolin administration to the D-gal group notably reversed these parameters with insignificant differences versus the Ctrl group. These results documented that luteolin could recover the D-gal-induced cognitive competencies in rats (Table 2).

**Table 2:** Effect of luteolin treatment on behavioural tests in all the studied groups

Group/parameters	Morris water maze test		Open field test	
	Escape latency (s)	No. of platform crossing	Total rearing / 5 min	Square crossed in 5 min
Group I (Ctrl)	11.7 ± 1.27	3.9 ± 0.69	16.1 ± 2.39	62.3 ± 7.21
Group II (Luteolin)	11.33 ± 1.32	3.8 ± 0.63	16.5 ± 2.37	63.9 ± 7.37
Group III (D-gal)	20.1 ± 2.42^*^	1.5 ± 0.53^*^	8.5 ± 1.58^*^	37.9 ± 4.89^*^
Group IV (D-gal/luteolin)	12.3 ± 1.89^#^	3.4 ± 0.52^#^	15.1 ± 2.28^#^	60.1 ± 5.81^#^

Data represents mean ± SD. Each group had *n* = 10 rats. P value < 0.05 was considered significant. ^*^ Significant difference of group (III) vs. group (I) (*P* < 0.05). ^***#***^ Significant difference of group (IV) vs. group (III) (*P* < 0.05)

### Effect of Luteolin Treatment on Body Weights and Brain Indices in D-Gal-Evoked Brain Ageing

**Fig. 2:**
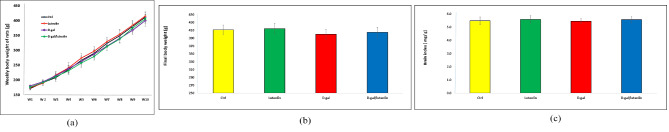
Effect of luteolin treatment on weekly body weights (**A**), final body weight (**B**), and brain indices (**C**) in all the studied groups. Data represents mean ± SD. Each group had *n* = 10 rats

Concerning the body weights of all rats, they were basically the same at the beginning of the experiment. At week 10, the final body weights of the D-gal group were slightly lower than those of the Ctrl one. In addition, the treated D-gal/luteolin group displayed a slight increase in body weights compared to the D-gal group. Nevertheless, there were no significant differences between all four groups, as shown in Fig. 2A-B. The same was noticeable for the rats’ brain indices; a slight diminution in the D-gal group was noticeable, meanwhile, there were insignificant differences between all tested groups, as displayed in Fig. 2-C.

### Effect of Luteolin Treatment on Cholinergic Functions and BDNF Relative Gene

**Fig. 3:**
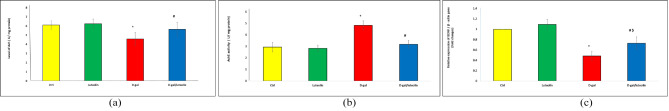
Effect of luteolin treatment on cholinergic functions and BDNF relative gene expression in D-gal-induced brain ageing. (**A**): Level of Ach (**B**): AchE activity (**C**): Relative expression of BDNF. Data represents mean ± SD. Each group had *n* = 10 rats. P value < 0.05 was considered significant. ACh, acetylcholine; AChE, acetylcholine esterase; BDNF, Brain-derived neurotrophic factor. ^*^ Significant difference of group (III) vs. group (I) (*P* < 0.05). ^***#***^ Significant difference of group (IV) vs. group (III) (*P* < 0.05). ^***$***^ Significant difference of group (IV) vs. group (I) (*P* < 0.05)

As depicted in Fig. 3A-B, the hippocampal Ach level was obviously lower, while AchE activity was higher in the D-gal group than the Ctrl one. It was noteworthy that the D-gal/luteolin co-treatment significantly reversed these cholinergic abnormalities, with an insignificant difference versus the Ctrl group. These results verified that D-Gal-induced hippocampal cholinergic dysfunction could be rescued by luteolin treatment.

In addition, D-gal treatment significantly downregulated BDNF gene expression compared to the Ctrl group. In contrast, luteolin/D-gal co-administration mitigated the D-gal-mediated BDNF downregulation in the treated group, yet it is still significantly lower than in the Ctrl group (Fig. 3-C).

### Effect of Luteolin Treatment on GLO1/AGE/RAGE Aaxis in D-Gal-Triggered Brain Aging

**Fig. 4:**
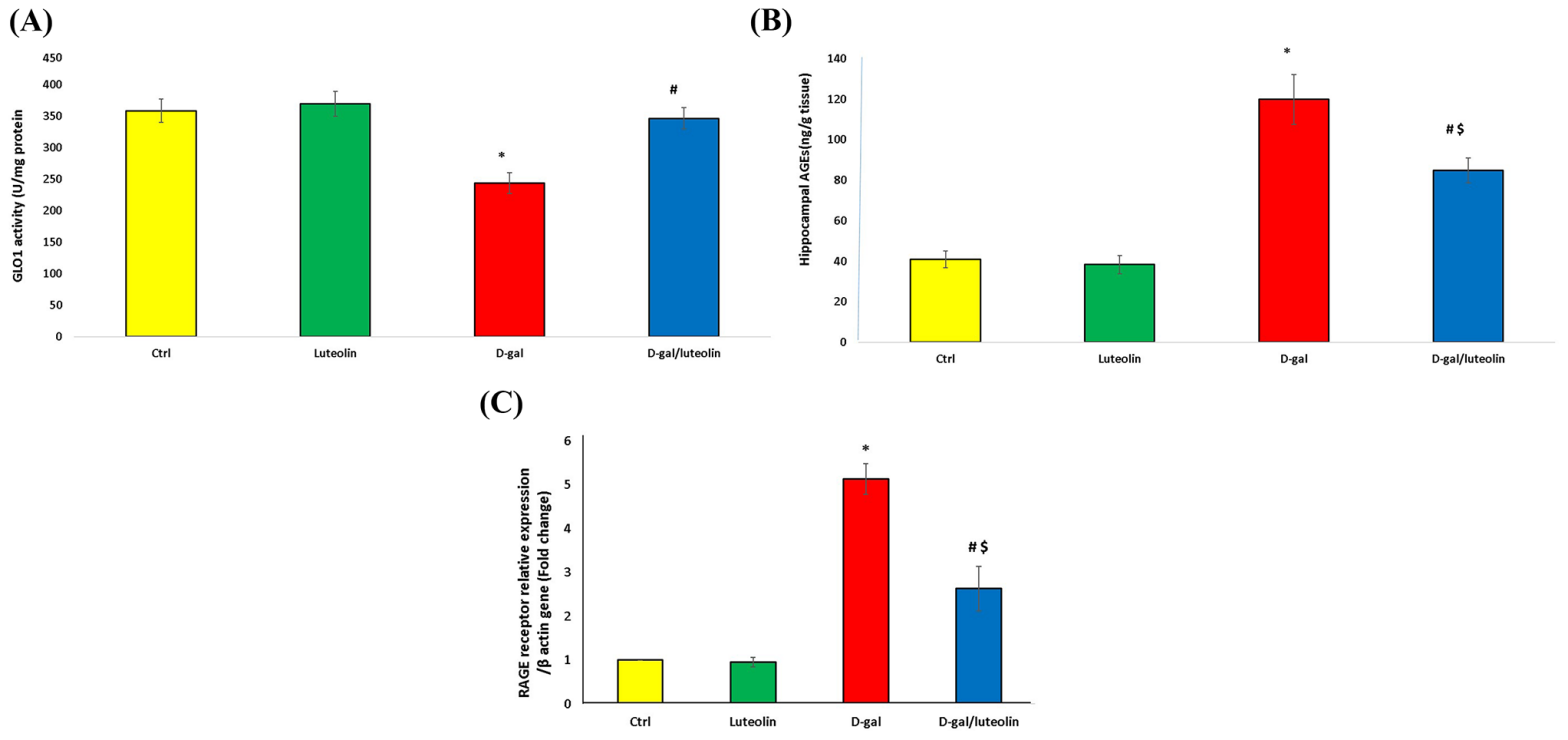
Effect of luteolin treatment on GLO1/AGEs/RAGE axis in brain tissues. (**A**) GLO1 activity (U/mg protein); (**B**) AGEs level (ng/g tissue); and (**C**) RAGE relative gene expression. Data represents mean ± SD. Each group had *n* = 10 rats. P value < 0.05 was considered significant. GLO1: glyoxalase 1; AGEs: advanced glycation end products; RAGE: receptor for AGE. ^*^ Significant difference of group (III) vs. group (I) (*P* < 0.05). ^***#***^ Significant difference of group (IV) vs. group (III) (*P* < 0.05). ^***$***^ Significant difference of group (IV) vs. group (I) (*P* < 0.05)

GLO1 activity was considerably decreased, while AGEs levels and RAGE mRNA expression were greatly elevated in the D-gal group compared with the Ctrl one. Importantly, luteolin administration effectively attenuated the D-gal-induced changes of these parameters in the D-gal/luteolin group compared to the D-gal only group, yet these levels are still significantly higher versus the Ctrl group except for GLO1 activity, which was insignificant versus the Ctrl group (Fig. 4A - D).

### Effect of Luteolin Treatment on Brain Redox Status, Neuro-Inflammatory, Apoptotic, and Senescence Markers

Results of Table 3 revealed that D-gal consecutively evoked a significant increment in hippocampal MDA level and XO enzyme activity; the opposite was true for SOD activities after D-gal exposure for 10 weeks. On the contrary, luteolin intervention could significantly reverse all of these parameters in the D-gal/luteolin group compared to the D-gal group, documenting its potent antioxidant criterion.

Here, D-gal-treated rats significantly overexpressed inflammation-related cytokines (IL-1β and TNFα) compared to Ctrl ones. However, D-gal/luteolin co-administration alleviated the D-gal-induced elevation of these inflammatory cytokines (Table 3***).*** This highlighted the anti-inflammatory role of luteolin on the D-gal-induced inflammation in this study.

Moreover, Table 3 showed that D-gal administration notably upregulated caspase-3 expression and p21 levels in the aging rats compared with the controls. By supplementing luteolin, caspase-3 upregulation and p21 levels were dramatically attenuated and insignificant versus the Ctrl group. These results revealed that luteolin could inhibit the D-gal-induced hippocampal apoptosis and cellular senescence, which could partly mediate its neuroprotection.

**Table 3:** Effect of luteolin treatment on hippocampal redox status, neuro-inflammatory, apoptosis, and senescence markers in rat’s hippocampal tissues

Parameters	Group I (Ctrl)	Group II (Luteolin)	Group III (D-gal)	Group IV (D-gal/luteolin)
MDA level (nmol/g tissues)	48.9 ± 5.22	44.2 ± 4.61	148.1 ± 10.88 ^*^	65.8 ± 6.30 ^# $^
XO activity (U/mg protein	0.0048 ± 0.0006	0.0043 ± 0.0006	0.0059 ± 0.0007 ^*^	0.0050 ± 0.0005 ^#^
SOD activity (U/mg protein)	1.79 ± 0.11	1.83 ± 0.09	0.62 ± 0.05 ^*^	1.74 ± 0.09 ^#^
TNF-α level (pg/mg protein)	16.4 ± 2.15	15.7 ± 2.41	32 ± 4.19 ^*^	20.9 ± 3.07 ^#$^
IL-1β level (pg/mg protein)	42.9 ± 5.24	40.2 ± 4.05	99.4 ± 8.73 ^*^	52.1 ± 5.15 ^# $^
Caspase 3 mRNA levels	1 ± 0	0.94 ± 0.07	1.871 ± 0.10 ^*^	1.072 ± 0.14 ^#^
P21 level (pg/mg protein)	5.32 ± 0.42111	4.94 ± 0.39	17.56 ± 1.56 ^*^	7.254 ± 0.68 ^# $^

Data represents mean ± SD. Each group had *n* = 10 rats. P value < 0.05 was considered significant. MDA, malondialdehyde; XO, xanthine oxidase; SOD, superoxide dismutase; TNF-α, tumour necrosis factor-α; IL-1β, interleukin-1β. ^*^ Significant difference of group (III) vs. group (I) (*P* < 0.05). ^***#***^ Significant difference of group (IV) vs. group (III) (*P* < 0.05). ^***$***^ Significant difference of group (IV) vs. group (I) (*P* < 0.05)

### Effect of Luteolin Treatment on Mitochondrial Function Markers

**Fig. 5:**
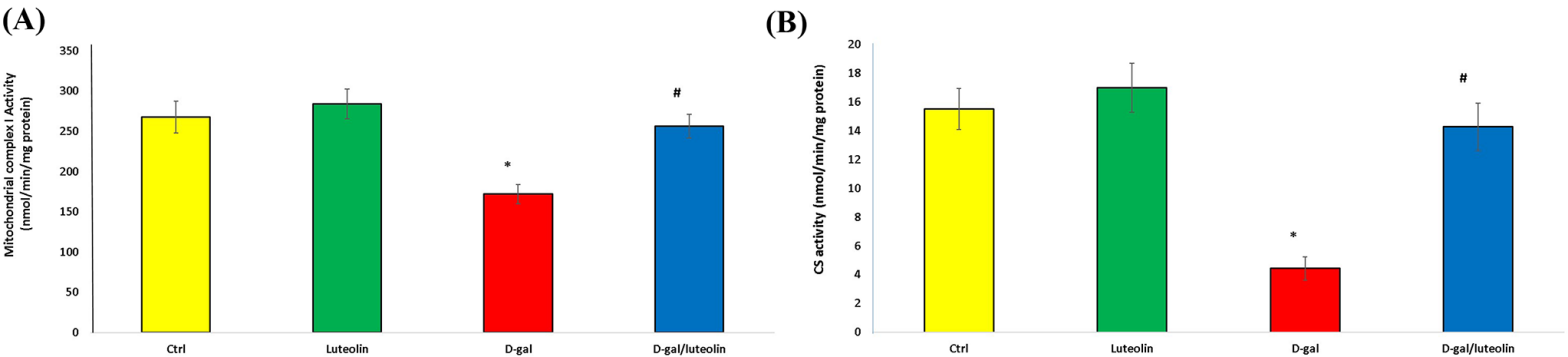
Effect of luteolin treatment on mitochondrial function markers in D-gal-evoked brain ageing. (**A**) Mitochondrial complex 1 activity (**B**) CS activity. Data represents mean ± SD. Each group had *n* = 10 rats. P value < 0.05 was considered Significant. CS; citrate synthase. ^*^ Significant difference of group (III) vs. group (I) (*P* < 0.05). ^***#***^ Significant difference of group (IV) vs. group (III) (*P* < 0.05)

A large body of evidence suggests that OS is linked with mitochondrial dysfunction in the ageing process [3]; therefore, we assessed whether luteolin treatment could rescue the d-gal-evoked aberrant mitochondrial function. In the present work, we measured the hippocampal mitochondrial complex I and CS Activity. As expected, both parameters scored a significant diminution in the d-gal-induced ageing group compared with the Ctrl one. Interestingly, the D-gal /luteolin cotreatment notably elevated these parameters with an insignificant difference versus the Ctrl group. This documented the potential role of luteolin in attenuating ageing-associated mitochondrial dysfunction in rats, as shown in Fig. 5A-B.

### Effect of Luteolin Treatment on Hippocampal SIRT1 mRNA Expression Levels in Brain Tissues

**Fig. 6:**
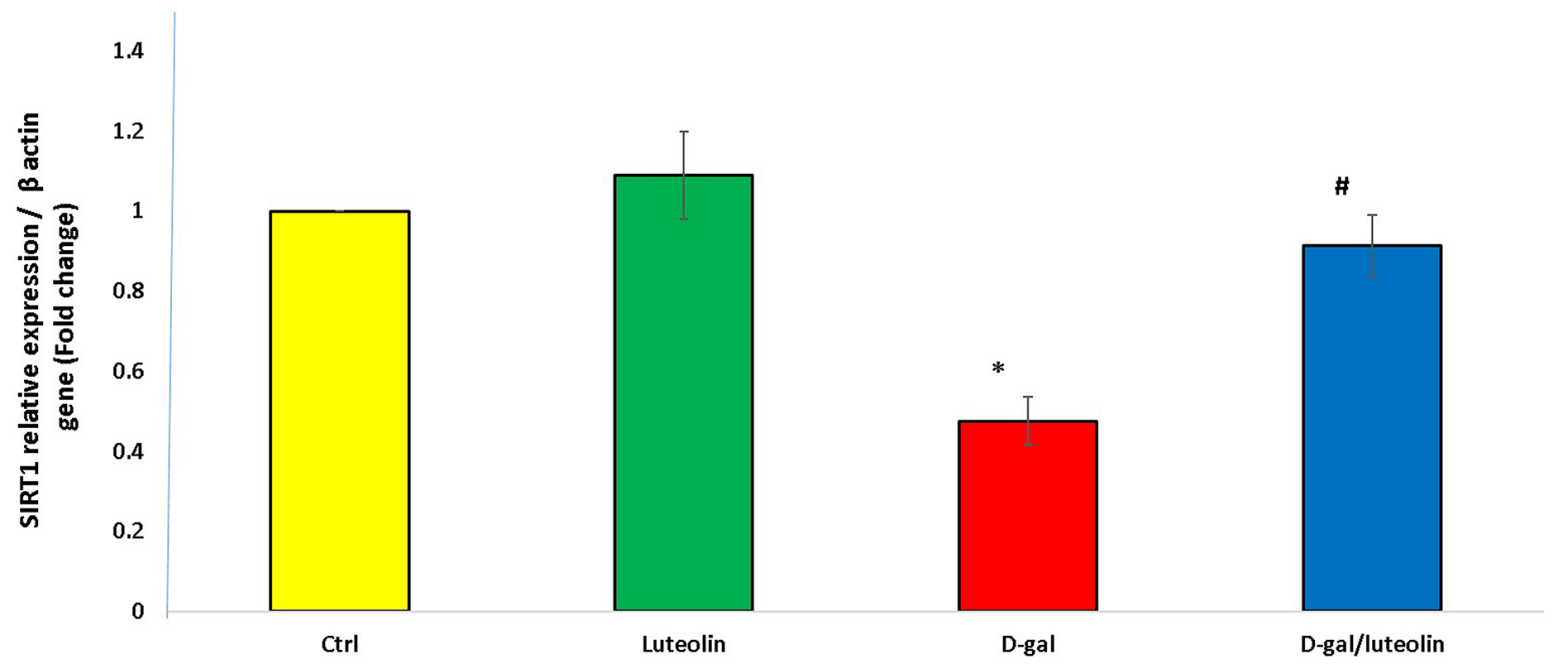
Effect of luteolin treatment on hippocampal SIRT1 mRNA levels. Data represents mean ± SD. Each group had *n* = 10 rats. P value < 0.05 was considered significant. SIRT1; sirtuin1. ^*^ Significant difference of group (III) vs. group (I) (*P* < 0.05). ^***#***^ Significant difference of group (IV) vs. group (III) (*P* < 0.05)

Considering that SIRT1 plays important roles in OS, inflammation, apoptosis, neuronal survival, and synaptic plasticity, we examined the gene expression level of this protein in the hippocampi of all tested groups. As depicted in Fig. 6, the expression level of SIRT1 was distinctly lower in the D-gal only group than in the Ctrl one. On the contrary, the D-gal/luteolin group displayed a significant upregulation of SIRT1 expression levels compared to the D-gal group. Further, the values of the D-gal/luteolin group showed an insignificant difference versus the Ctrl one.

### Correlations Between SIRT1 Relative Expression and Other Studied Parameters in Group III (D-Gal) and Group ΙV (D-Gal/Luteolin)

Table 4 revealed that, in the D-gal group (group III) and the D-gal/luteolin (group IV), SIRT1 was positively correlated with the hippocampal Ach level and BDNF relative expression. In addition, SIRT1 showed a positive correlation with the GLO1, SOD, mitochondrial complex I and CS activity in both groups. Conversely, significant negative correlations were noticed between SIRT1 and hippocampal AchE and XO activity alongside the levels of MDA, TNF-α, IL-1β, AGEs, and P21. Additionally, SIRT1 was negatively correlated with the relative gene expression of RAGE and caspase 3 in both groups.

**Table 4:** Correlations between SIRT1 relative expression and other studied parameters in group III (D-gal) and group ΙV (D-gal/luteolin)

Parameters	SIRT1 expression	
	Group III (D-gal) *r* value	Group IV (D-gal/luteolin) *r* value
GLO1 activity (U/mg protein)	0.895^*^	0.842^*^
AGEs	− 0.898^*^	-0.784
RAGE relative expression	− 0.849^**^	− 0.756^*^
P21 (pg/mg protein)	− 0.804^*^	− 0.718^*^
BDNF relative expression	0.880^*^	0.892^*^
Ach level (U/mg protein)	0.677*	0.880*
AchE activity (U/mg protein)	− 0.950^*^	− 0.892^*^
MDA level (nmol/g tissues)	− 0.935^*^	− 0.920^*^
XO activity (U/mg protein)	− 0.877^*^	− 0.727^*^
SOD activity (U/mg protein)	0.880^*^	0.779^*^
TNF-α level (pg/mg protein)	− 0.691^*^	− 0.722^*^
IL-1β level (pg/mg protein)	− 0.902^*^	− 0.634^*^
Mitochondrial complex I Activity (nmol/min/mg protein)	0.900^*^	0.710^*^
CS activity (nmol/min/mg protein)	0.951^*^	0.709^*^
Caspase 3 relative expression	− 0.901^*^	− 0.817^*^

^*^Statistically significant at *P* < 0.05

### Effects of Luteolin Treatment on Hippocampal Histological Structure in D-Gal-Evoked Brain Ageing

**Fig. 7:**
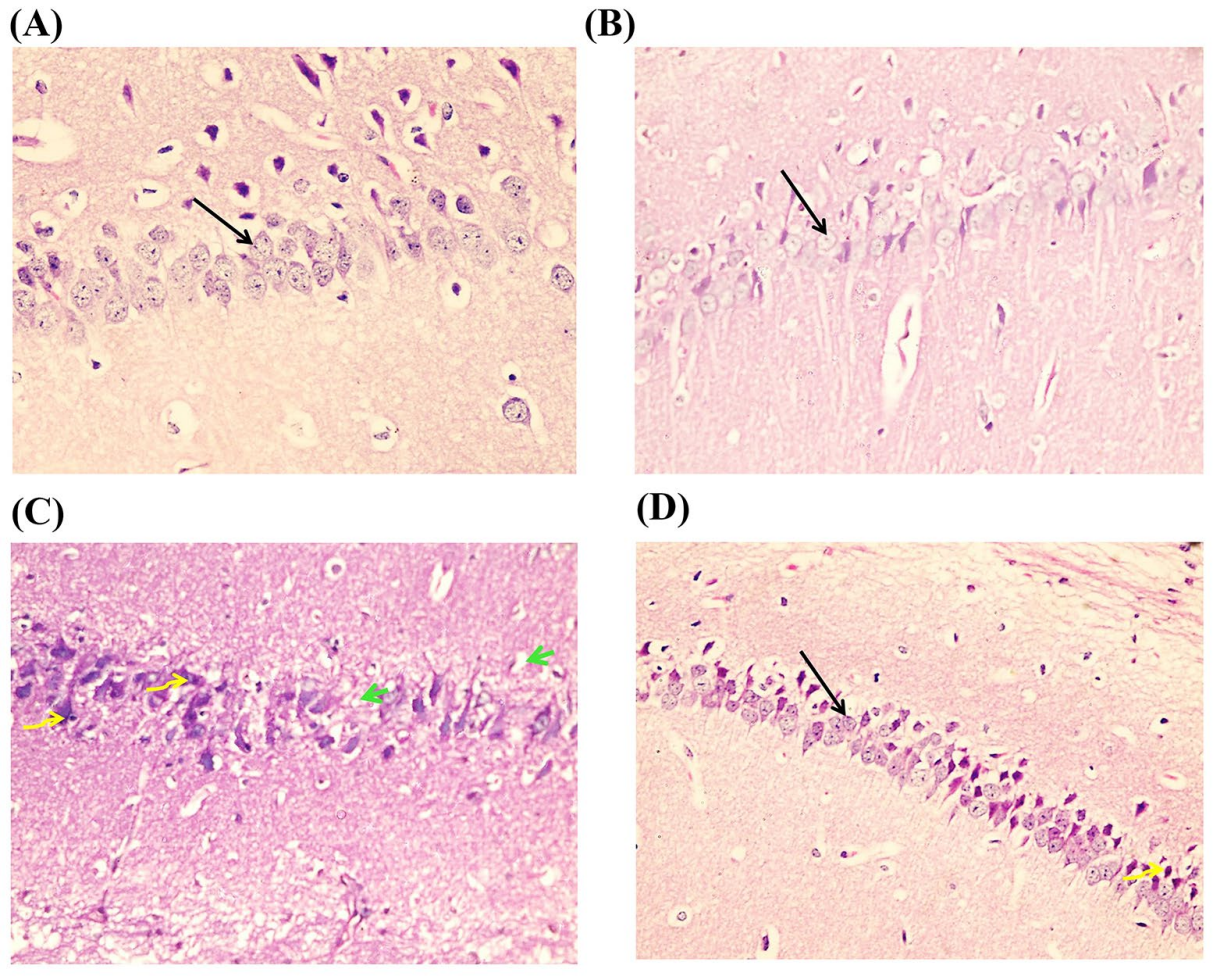
Photomicrographs of CA1 of hippocampus of the four studied groups with H & E (× 400). The CA1 region in the control group Fig. 7(**A**) and luteolin treatment Fig. 7(**B**) consist of pyramidal cells with rounded vesicular nuclei, prominent nucleoli, and apical dendrites (arrows). In the D-gal group Fig. 7(**C**), pyramidal cells show darkly stained pyknotic nuclei (curved arrows) and some areas are devoid of cells (short green arrows). D-gal/luteolin co-treatment significantly reserved the normal architecture of the CA1 region Fig. 7 (**D**) with appearance of normal pyramidal (arrows) and some pyknotic cells are also seen (curved arrows). (Bar:50 μm)

**Fig. 8:**
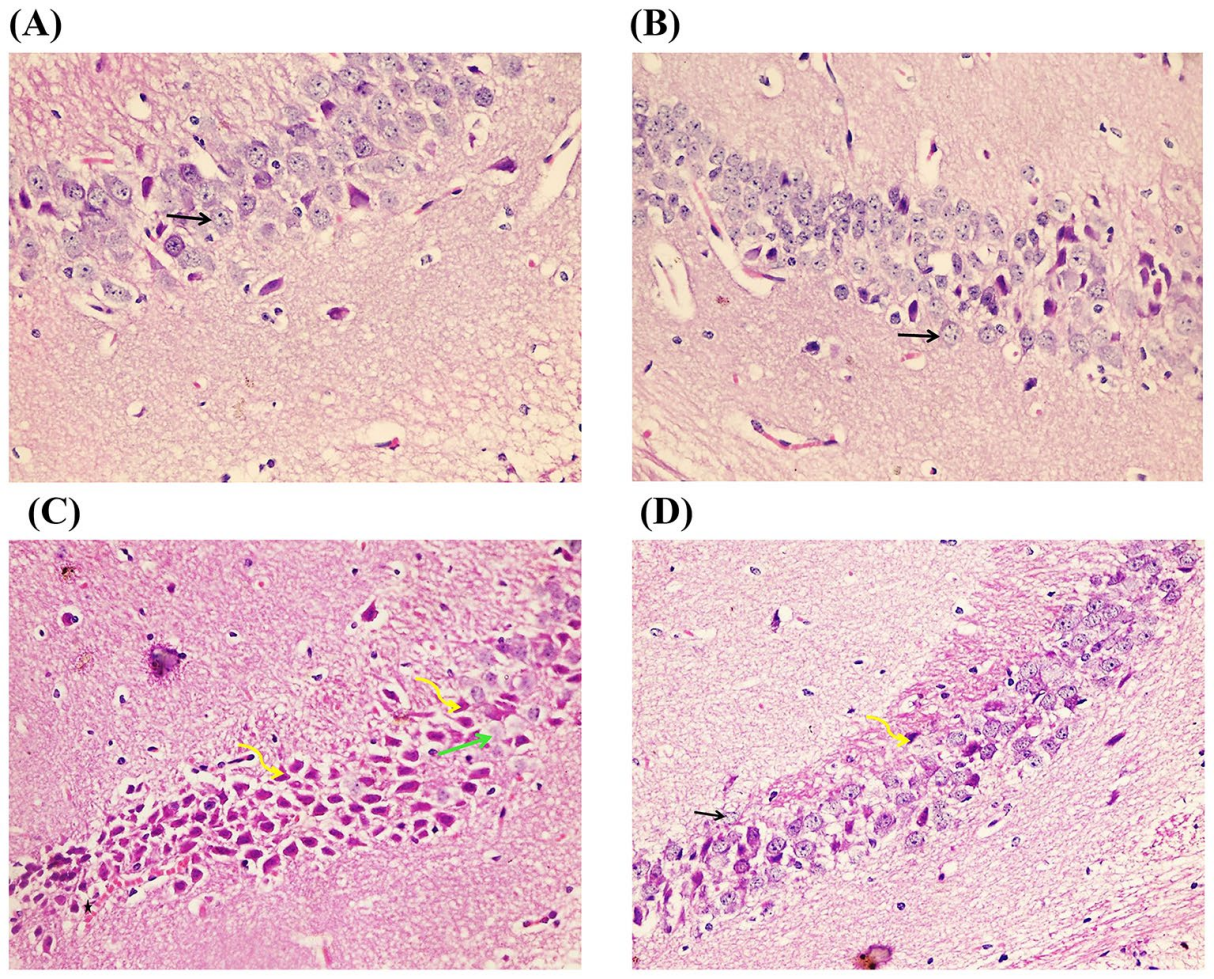
Photomicrographs of CA3 of hippocampus of the four studied groups with H & E (× 400). The CA3 region in the control group Fig. 8 (**A**) and luteolin treatment Fig. 8 (**B**) consist of 3–4 rows of pyramidal cells with rounded vesicular nuclei and prominent nucleoli (arrows). In the D-gal group Fig. 8 (**C**), most of the pyramidal cells appear darkly stained with pyknotic nuclei (curved arrows), some cells show karyolitic nuclei (green arrow) and some areas show haemorrhage (star). D-gal/luteolin co-treatment significantly reserved the normal architecture of the CA3 region Fig. 8 (**D**) with the appearance of normal pyramidal (arrows) and some pyknotic cells are also seen (curved arrows). (Bar:50 μm)

The CA1 region of the hippocampus in control and luteolin treatment groups showed normal architecture in the form of # pyramidal cells with rounded vesicular nuclei, prominent nucleoli and apical dendrites. In the D-gal group, pyramidal cells showed a loss of normal architecture with darkly stained pyknotic nuclei, and some areas were devoid of cells. D-gal/luteolin co-treatment significantly reserved the normal architecture of the CA1 region with appearance of normal pyramidal, but few pyknotic cells were still seen (Fig. 7). CA3 region in the control group and luteolin treatment consisted of 3–4 rows of pyramidal cells with rounded vesicular nuclei and prominent nucleoli. In the D-gal group, most of the pyramidal cells appear darkly stained with pyknotic nuclei, some cells show karyolitic nuclei, and some areas show haemorrhage. D-gal/luteolin co-treatment significantly reserved the normal architecture of the CA3 region with appearance of normal pyramidal, and some pyknotic cells were also seen (Fig. 8).

### Effects of Luteolin Treatment on Hippocampal Immunohistological Studies

**Fig. 9:**
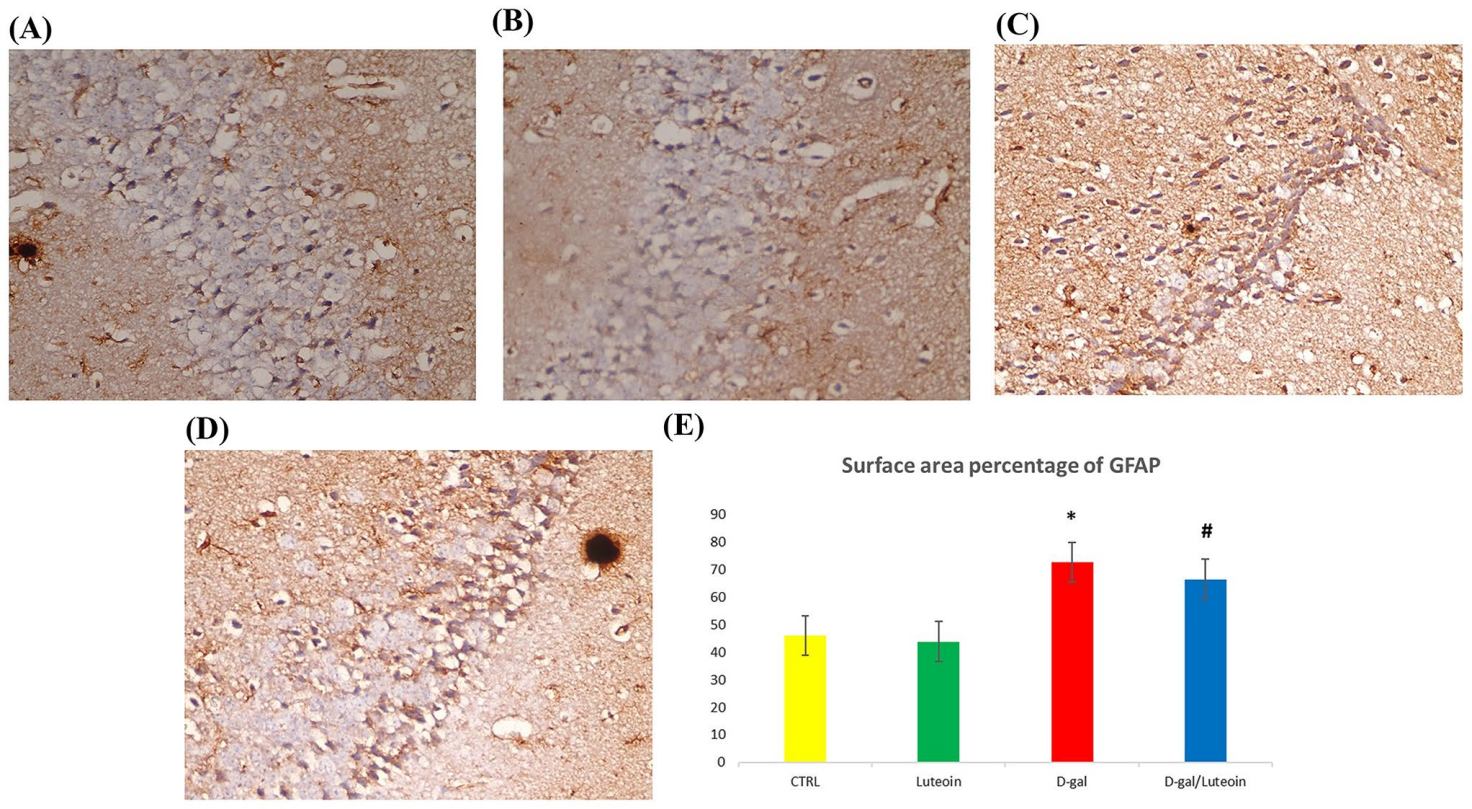
Photomicrographs of hippocampus of the four studied groups with GFAP (× 400). In the D-gal group Fig. 9 (**C**), marked elevation in the surface area percentage of GFAP is noticed (Brown colouration) as compared with the control group Fig. 9 (**A**) and luteolin treatment Fig. 9 (**B**). D-gal/luteolin co-treatment significantly decreased the surface area percentage of GFAP Fig. 9 (**D**) as compared with the D-gal group (Bar: 50 μm). Figure 9 (**E**): Statistical analysis of surface area % of GFAP. Data represents mean ± SD. Each group had *n* = 10 rats. P value < 0.05 was considered. Significant. ^*^ Significant difference of group (III) vs. group (I) (*P* < 0.05). ^***#***^ Significant difference of group (IV) vs. group (III) (*P* < 0.05)

GFAP immunostained sections of the hippocampus CA3 region and statistical analysis of morphometric studies of surface area % of GFAP revealed that in the D-gal group, there was a marked elevation in the surface area percentage of GFAP as compared with Ctrl and luteolin groups. In the D-gal/luteolin co-treatment group, the surface area percentage significantly increased as compared with the control and Luteoin groups but significantly decreased as compared with the D-gal group (Fig. 9).

**Fig. 10:**
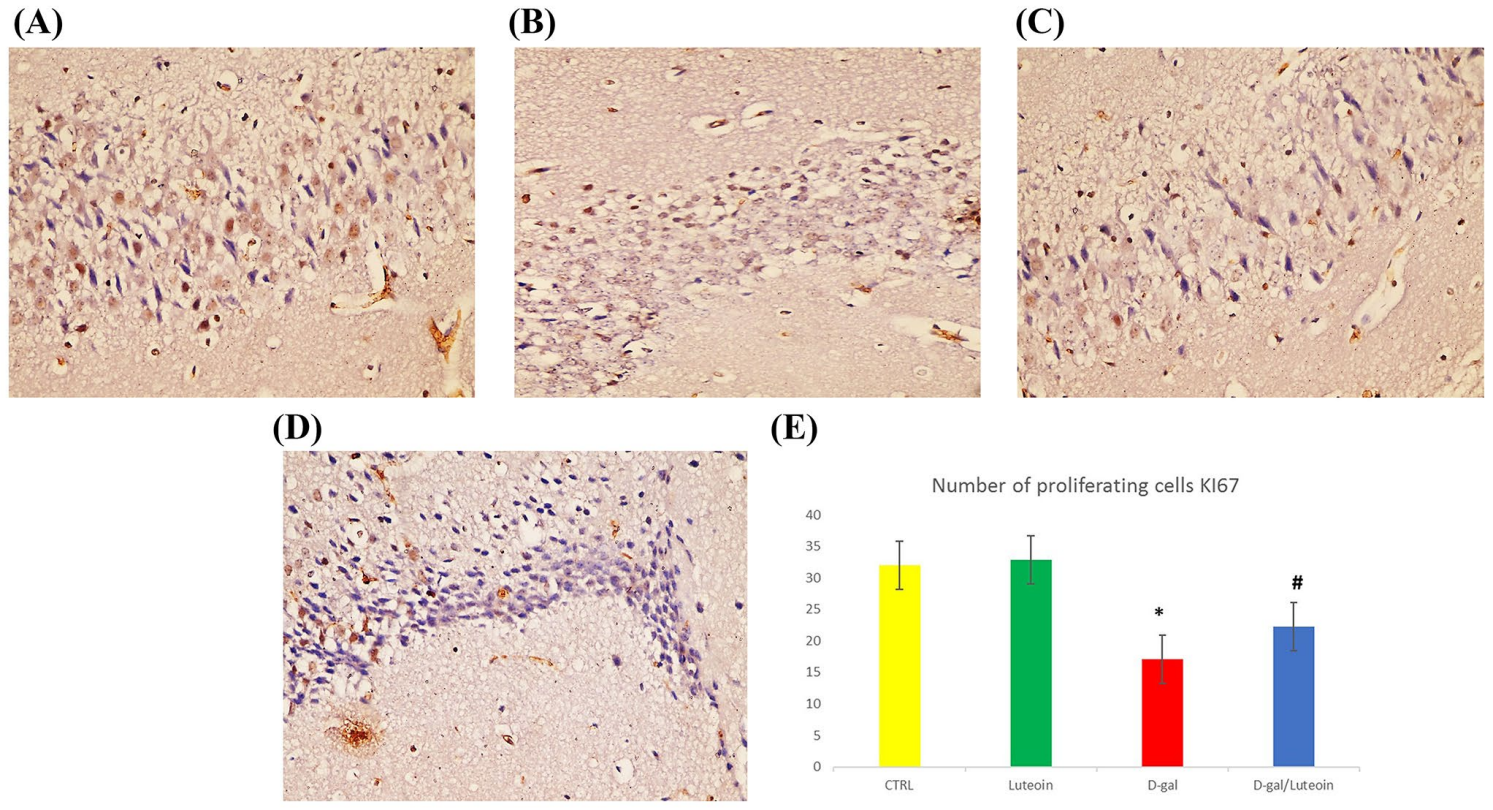
Photomicrographs of hippocampus of the four studied groups with Ki67 (× 400). D-gal group Fig. 10 (**C**) show a marked reduction in the number of proliferating cells (Brown nuclei) as compared with the control group Fig. 10(**A**) and luteolin treatment Fig. 10(**B**). D-gal/luteolin co-treatment significantly increased the number of proliferating cells Fig. 10 (**D**) as compared with D-gal group Fig. 10 (**C**). (Bar: 50 μm). Figure 10 (**E**): Statistical analysis of number of proliferating cells. Data represents mean ± SD. Each group had *n* = 10 rats. P value < 0.05 was considered significant. *Significant difference of group (III) vs. group (I) (*P* < 0.05). ^#^ Significant difference of group (IV) vs. group (III) (*P* < 0.05)

Examination of Ki67 immunostained sections of the hippocampus CA3 region and statistical analysis of the number of proliferating cells revealed a marked reduction in the number of proliferating cells in the D-gal group as compared with the Ctrl group and luteolin treatment. D-gal/luteolin co-treatment significantly increased the number of proliferating cells as compared with the D-gal group (Fig. 10).

## Discussion

As a study novelty, luteolin’s neuroprotective properties in a model of brain ageing in rats and its potential molecular mechanisms have been demonstrated. This is the first study to implicate SIRT1 as a crucial upstream molecule that mediate luteolin’s modulation of GLO1/AGE/RAGE signalling and its associated neutotoxicity, involving OS, mitochondrial dysfunction, neuro-inflammation, apoptosis, and senescence.

According to our study, luteolin effectively ameliorated brain agieng as it enhanced cognative function and cholinergiac transmission and additionally improved the cytomorhological alterations in the hippocampal regions of the D-gal group. This came in line with He et al. [16] and Ahmad et al. [19], who validated the neuroprotective impact of luteolin.

In the present work, the MWM test revealed an evident impairment in both short and long-term memories among D-gal rats, which was in harmony with previous findings. [1] Conversely, luteolin treatment efficiently improved rats’ spatial memory, as indicated by the diminished escape latency and increment in the number of platform crossings. Furthermore, outcomes from the OFT demonstrated the notable competence of luteolin in enhancing the rats’ exploratory capabilities in a novel environment. Collectively, these findings suggested that luteolin has the potential to serve as a protective agent against D-gal-triggered memory impairment.

Certainly, Ach is a crucial neurotransmitter, implicated in learning and memory, and regulated by the AchE enzyme. Cholinergic neurotransmission has been improved by activating cholinergic receptors and elevating the amount of Ach in the neuronal synaptic cleft [1]. Dynamic alterations in Ach and AchE activity are linked to progressive cognitive deterioration [34]. The neurotrophic factor BDNF is a crucial molecular mediator in synaptic transmission, plasticity, and neuronal proliferation. Furthermore, BDNF enhances the viability and differentiation of cholinergic neurons and induces the release of Ach, thereby essential for learning and memory. [35] During ageing, the cholinergic neurons exhibit neurodegenerative changes, highlighted by decreased choline acetyltransferase and raised AchE activities, ultimately reducing Ach release. Likewise, BDNF levels decrease considerably in ageing, contributing to cognitive deficits. [36]

Consistently, chronic D-gal administration herein dramatically boosted AchE activity and substantially reduced Ach and BDNF levels in rats’ brains. The increased AchE enzymatic activity caused by D-gal-associated OS was shown to be strongly correlated with learning decline and memory deficits, as affirmed previously [1], which agreed with our findings. Importantly, luteolin treatment herein significantly restored the normal levels of BDNF and Ach and lowered the AchE activity, which in turn could enhance the cholinergic neurotransmission and cognitive capabilities. These data highlighted the neuroprotective impact of luteolin in this ageing model, which is highly consistent with Ryu et al. [37] and Ali et al. [38]

The physiological levels of GLO1 have been postulated to regulate organismal physiology and may offer novel targets for postponing ageing and associated illnesses. Our results revealed a significant diminution in hippocampal GLO1 activity with concomitant elevated AGEs levels and RAGE expression levels in the D-gal-treated rats. In harmony, Li et al. [13] showed that long-term D-gal administration reduced GLO1 and increased MG accumulation, the primary precursor to intracellular AGEs. The augmented AGEs enhanced the expression of their specific receptors, RAGE, which bound to AGEs in a dose-dependent manner [6, 39]. Moreover, Faruqui et al. [40] stated that the AGE-RAGE interactions and binding of RAGE with various other ligands not only contribute to the exacerbation of OS but also to the over-expression of RAGEs themselves. AGE/RAGE signalling activation triggered OS, mitochondrial dysfunction, chronic inflammation, and apoptosis, with subsequent brain senescence [8], all of which were proved and mentioned later herein by our findings.

Surprisingly, luteolin intervention induced the activities of the GLO1 enzyme, which could be attributed to its SIRT1 upregulation as delineated later by our findings. Activation GLO1 enzyme has been implicated in MG detoxification and counteracting D-gal-induced oxidative damage; this is supported herein by the notable reduction of AGEs and downregulation of RAGE upon luteolin co-treatment. In line, the AGEs/RAGE transduction axis was downregulated by luteolin in an earlier study. [19]

Based on previous reports, including our findings, the binding of AGEs to RAGE triggers OS, activating the RAGE signal pathway and contributing to the aetiopathogenesis of D-gal-induced oxidative brain damage [41]. A manifest elevated MDA level and XO activity, while reduced SOD activities were observed in D-gal rats, all of which were reversed upon luteolin intervention. This coincides with the results of Alekhya Sita et al. [15] and Liu Y et al. [42], who acknowledged luteolin’s antioxidant potential. Our data highlighted that luteolin could alleviate the D-gal-evoked OS, making it a promising candidate for neurodegenerative disease prevention and treatment.

Interestingly, D-gal-provoked OS could trigger mitochondrial damage and dysfunction, which is a major cause of neurodegenerative disorders. [43] In support, D-gal intervention significantly reduced the energetic function of mitochondria as delineated herein by the reduced hippocampal mitochondrial complex I and CS Activities, which was in consistent with Liu et al.‘s [41] previous results. On the other hand, luteolin could successfully reverse the D-gal-evoked mitochondrial dysfunction, which agreed with previous findings. [16] This could be attributed to its antioxidant potential, hindering mitochondrial oxidative damage and decreasing the AGEs and their related neurotoxicity [43]. Furthermore, Hu et al. [44] reported that luteolin raised the mitochondrial membrane potential in LPS-treated cardiomyocytes and improved the CS activity, ATP content, and mitochondrial complex I/II/III/IV/V activities, documenting our findings.

Concomitantly, OS can trigger inflammatory responses in D-gal-induced brain injury. [45] In harmony, an obvious escalation in IL-1β and TNF‐α levels was noted in the D-gal-treated rats, pointing to hippocampal neuroinflammation, which was substantially reversed by luteolin treatment, proving its anti-inflammatory effect, which has been formerly observed in various studies. [46] .

It is common practice to use the astrocyte activation biomarker GFAP as a measure of reactive gliosis, a characteristic of the ageing brain. [47] Thus, the relationship between soluble cytokine release and activated astrocytes/microglial cells raises the assumption that inflammatory processes are important in the pathophysiology of ageing. [48] Immunohistochemical findings elucidated that luteolin administration decreased the elevated GFAP expression in the hippocampal regions of D-gal-induced ageing rats, suggesting that luteolin may prevent hippocampal senescence as supported previously. [49]

Interestingly, compelling evidence postulated that mitochondrial dysfunction, OS, and inflammation mechanisms work in harmony to induce apoptotic neuronal cell death in D-gal-induced brain injury. [50] Consistently, the current investigation revealed dramatic up-regulation of caspase-3 relative gene expression, the executioner of apoptosis, in the hippocampal region. In contrast, luteolin exhibited anti-apoptotic properties revealed via significantly downregulating hippocampal caspase 3 gene expression, preventing downstream executioner protease production, which coincides with previous reports. [51, 52]

Matched with Ma et al. [8], we speculated that brain senescence is the convergence point for the activated AGE/RAGE signalling, and its associated OS, mitochondrial dysfunction, inflammation, and apoptosis were shown to be cardinal factors in evoking brain senescence. In parallel, the D-gal rats exhibited a significant escalation in senescence-associated biomarker P21, which was shown to be a critical mediator of P53-dependent cell cycle arrest. [53] Meanwhile, luteolin administration substantially downregulated the AGEs/RAGE/P21 signalling pathway, which could be mediated via its induction to GLO1 enzyme activities, counteracting the D-gal-induced oxidative brain senescence. In support, Zhu et al.‘s [5] study suggested that luteolin suppresses cellular senescence induced by H_2_O_2_ in an oxidant-challenged model. To the author’s knowledge, this is the first study to reveal that luteolin’s modulation on the GLO1/AGEs/RAGE/P21 signalling pathway is a cardinal mediator for its senolytic criteria in this brain ageing model.

The ageing elicited upregulation of the p53/p21 pathway was suggested to stop the cell cycle and proliferation, as demonstrated herein by a notable decline in Ki67 (cellular proliferation biomarker) immunoreactivity in ageing rats. [54] Interestingly, our findings observed that luteolin was able to reverse the decline in Ki67 in the ageing rats, indicating enhanced hippocampal neurogenesis, which was in line with the former study of Zhou et al. [55] Luteolin’s antioxidant effect, as recorded herein, could contribute to maintain balance in the redox state of neurons, which promotes cell proliferation, growth, and differentiation. [56]

Intriguingly, SIRT1 is involved in memory, learning, cognitive function, and neural differentiation [57]. Matched with previous studies [5], our data strongly suggested that luteolin could significantly upregulate the SIRT1 expression level that was downregulated in the ageing rats. SIRT1 has been proven beneficial in neurodegenerative diseases by regulating their pathogenic mediators, involving OS, inflammation, mitochondrial dysfunction, cellular apoptosis, and senescence. [58] SIRT1 confers cellular protection against OS through numerous mechanisms, such as modulating forkhead transcription factors, enhancing catalase activity, and inducing manganese SOD. [59] Interestingly, SIRT1 is one of the signalling pathways that control the expression of peroxisome proliferator-activated receptor-γ coactivator-1α, which is the main regulator of mitochondrial biogenesis. [8] Emerging research indicates that SIRT1 is a potent inhibitor of NF-κB signalling, contributing to its anti-inflammatory signature. Furthermore, SIRT1 downregulates p21 via deacetylation and inactivation of its upstream p53. [13]

Importantly, SIRT1 was documented for its upregulation of GLO1 activity [60, 61], consequently decreasing AGEs accumulation with ultimate suppression of the RAGE signalling pathway. In the same line, Zeng et al., [62] reported downregulation of RAGE signaling by SIRT1 via NF-kB blockage-dependant mechanism. These data hypothesize SIRT1 as a crucial upstream molecule that mediates luteolin’s modulation of the GLO1/AGE/RAGE signalling pathway and its associated neurodegenerative pathology, making SIRT1 a pivotal mediator of luteolin’s neuroprotective potential in our model.

**Fig. 11:**
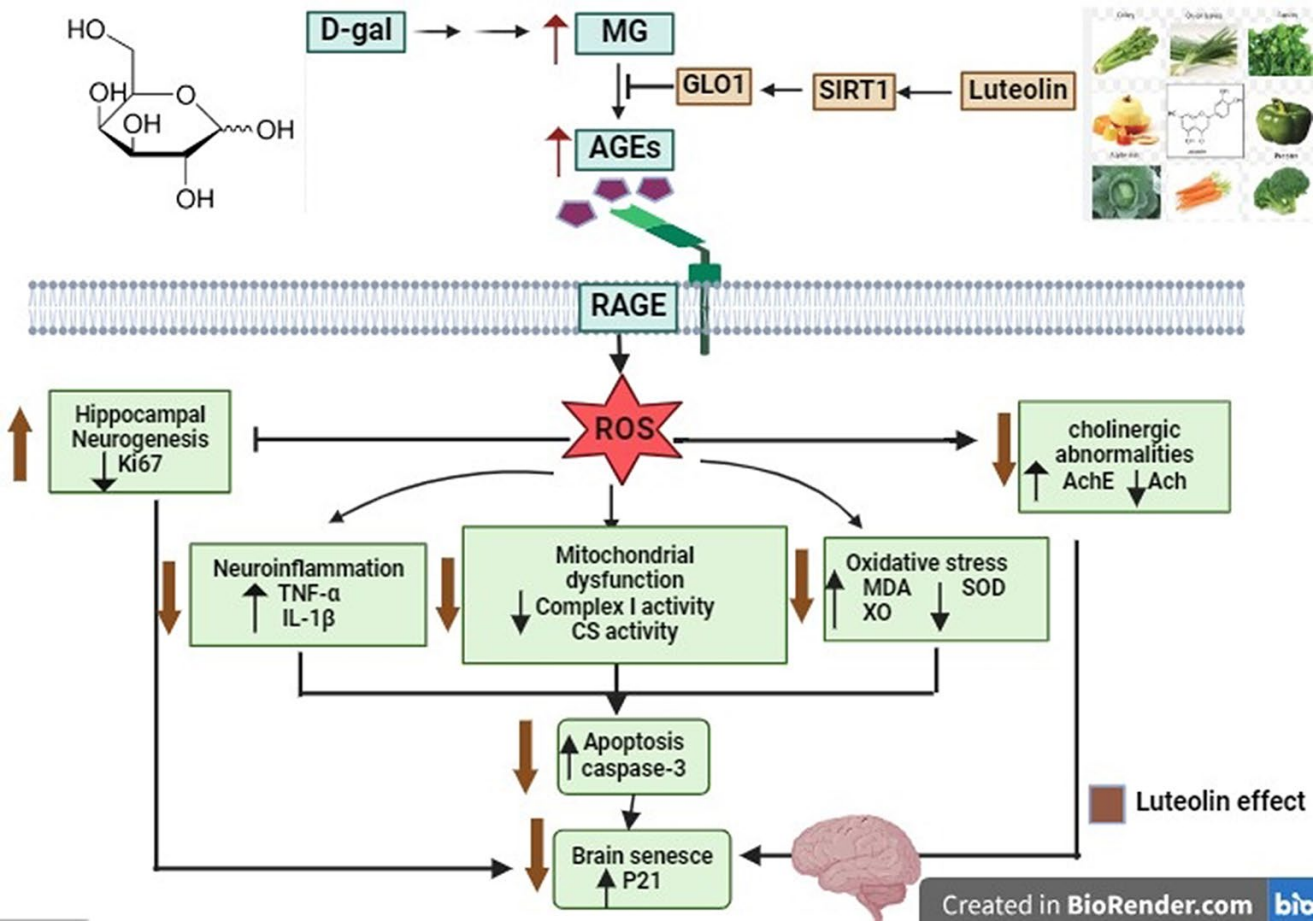
The proposed mechanisms implicated in luteolin’s neuroprotective potency against D-gal-induced brain ageing in rats

This was further confirmed by our correlation analysis, in which SIRT1 relative expression was positively correlated with the hippocampal levels of Ach level and BDNF along with GLO1, SOD, mitochondrial complex I and CS activities, while exhibiting negative correlations with hippocampal AchE and XO activities besides MDA, TNF-α, IL-1β, AGEs, and P21 levels. Collectively, we could speculate that luteolin protects neuronal tissue against D-gal-provoked OS, mitochondrial dysfunction, neuro-inflammation, and senescence, potentially via SIRT1 upregulation, which showed great relevance. However, additional research is required to identify the upstream regulatory factors involved in luteolin’s SIRT1 upregulation. Figure 11 illustrates the proposed mechanisms that underlie luteolin’s neuroprotective signature against D-gal-induced brain ageing in rats.

## Conclusion

Our study validated for the first time the efficacy of luteolin in mitigating D-gal-induced cognitive decline and hippocampal senescence. Luteolin scored comprehensive modulation of OS, mitochondrial dysfunction, neuro-inflammation, and neuronal apoptosis, coupled with promoting hippocampal neuroregeneration. The observed benefits are potentially mediated by luteolin’s upregulation of SIRT1 in the hippocampus, with subsequent modulation of the GLO1/AGE/RAGE signalling pathway and its associated neurodegenerative pathology. This study provides substantial theoretical support for the natural flavonoid luteolin’s use to prevent or treat ageing-related disorders.

## Data Availability

No datasets were generated or analysed during the current study.

## Abbreviations

D-gal: D-galactose
OS: Oxidative stress
AGEs: Advanced glycation end products
RAGE: Receptors advanced glycation end products
ROS: Reactive oxygen species
MG: Methylglyoxal
GLO: Glyoxalase
SIRT1: Sirtuin 1
NAD^+^: Nicotinamide adenosine dinucleotide
STAT3: Signal transducer and activator of transcription
PGC: Proliferator-activated receptor
MWM: Morris water maze
SC: Subcutaneous
IP: Intraperitoneal
DMSO: Dimethyl sulfoxide
OFT: Open field test
GSH: Glutathione
Ach: Acetylcholine
AChE: Acetylcholine esterase
DTNB: 5,5-dithiobis-2-nitrobenzoic acid
TBARS: Thiobarbituric acid reactive substances
TBA: Thiobarbituric acid
MDA: Malondialdehyde
XO: Xanthine oxidase
SOD: Superoxide dismutase
TNF-α: Tumour necrosis factor-α
IL: Interleukin
CS: Citrate synthase
ETC: Electron transport chain
GFAP: Glial fibrillary acidic protein
